# Supplementary material for: Improving root characterisation for genomic prediction in cassava
Source: Sci Rep. 2020 May 14;10:8003. doi: 10.1038/s41598-020-64963-9 (PMC7224197; doi:10.1038/s41598-020-64963-9)
Supplement: Supplementary file 8 — Supplementary information8. [file 41598_2020_64963_MOESM8_ESM.pdf]

Improving root characterisation for genomic prediction in cassava

Bilan Omar Yonis[1], Dunia Pino del Carpio[2,5**], Marnin Wolfe[2], Jean-Luc Jannink[2,3], Peter Kulakow[4], Ismail Rabbi[4*]**

[1] Montpellier SupAgro, 34060 Montpellier Cedex 02, France

[2] Department of Plant Breeding and Genetics, Cornell University, Ithaca NY 14850.

[3] US Department of Agriculture - Agricultural Research Service (USDA-ARS), Ithaca, NY.

[4] International Institute for Tropical Agriculture (IITA), Ibadan, Nigeria.

[5] Department of Jobs, Precincts and Regions, AgriBio, Centre for AgriBioscience, Bundoora, Australia

**equal contribution

*Corresponding author to I.Rabbi@cgiar.org

Supplementary Figures

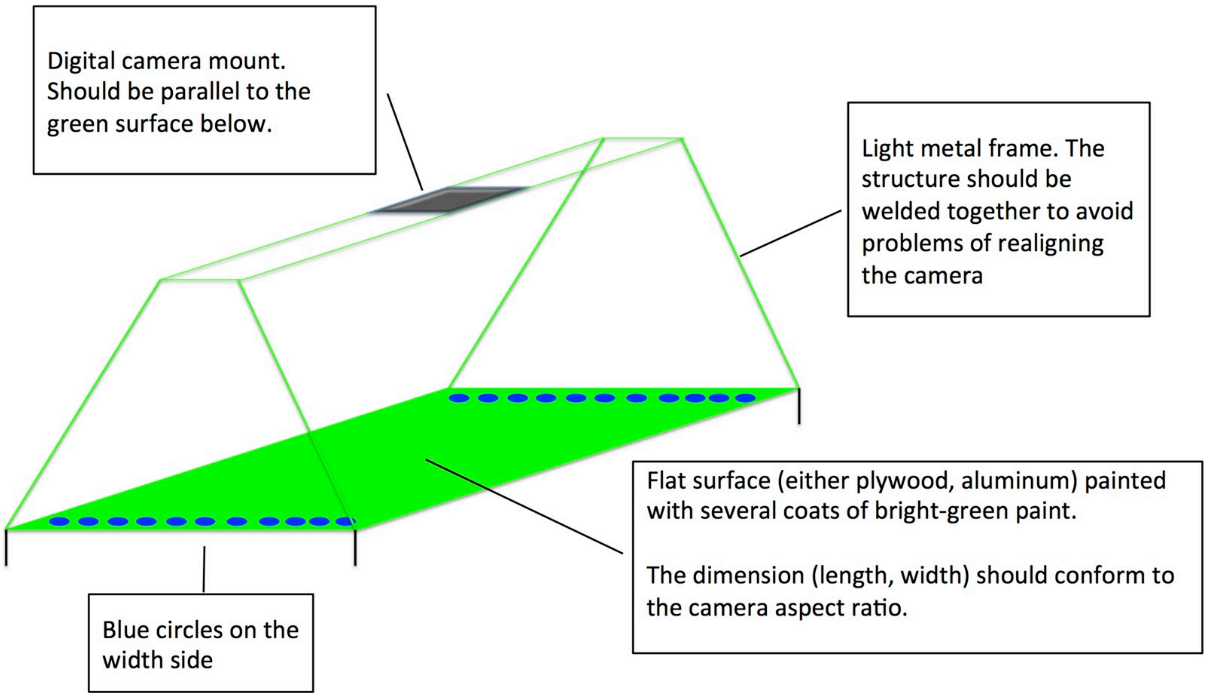

Supplementary figure 1: Schematic of the green board used as a background to take photographs in the field.

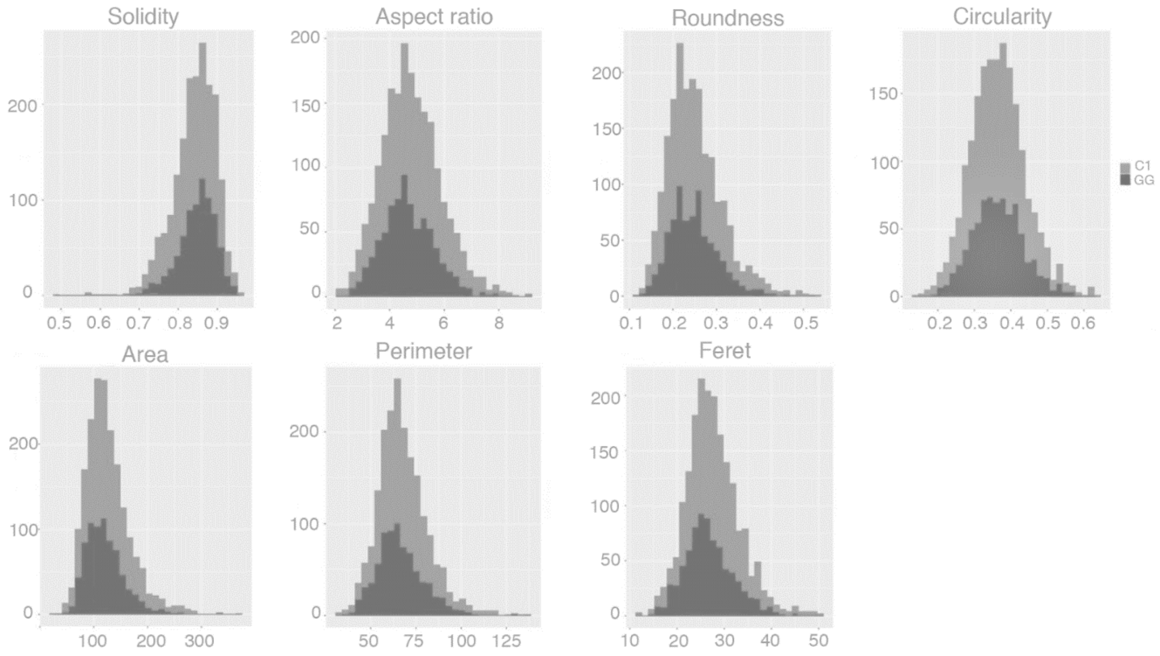

Supplementary Figure 2. Trait distribution of mean root values. Root values of each phenotype extracted from photographs from each genotype were averaged and the distribution plotted.Dark gray: GG, light gray : C1.

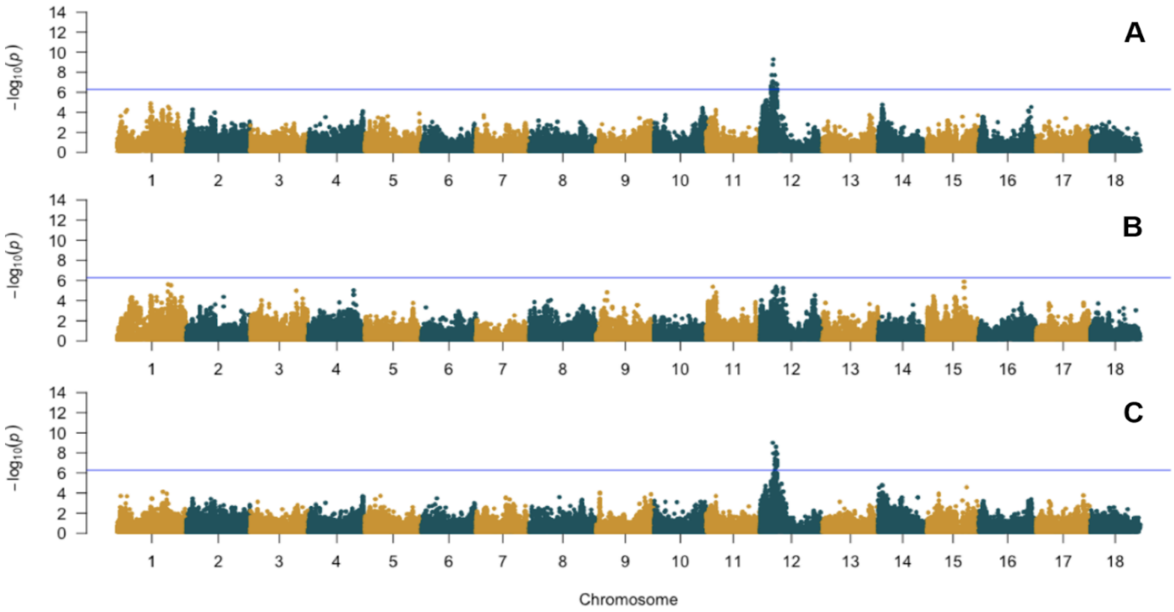

Supplementary figure 3. Yield traits GWAS results using the IITA Genetic Gain and Cycle 1 breeding populations. Manhattan plots of -$\log_{10}$(P-value) of A. Root weight, B. Shoot weight, C. Root number. Blue horizontal line indicate the Bonferroni statistical threshold (-$\log_{10}$(P-value) >6.28)

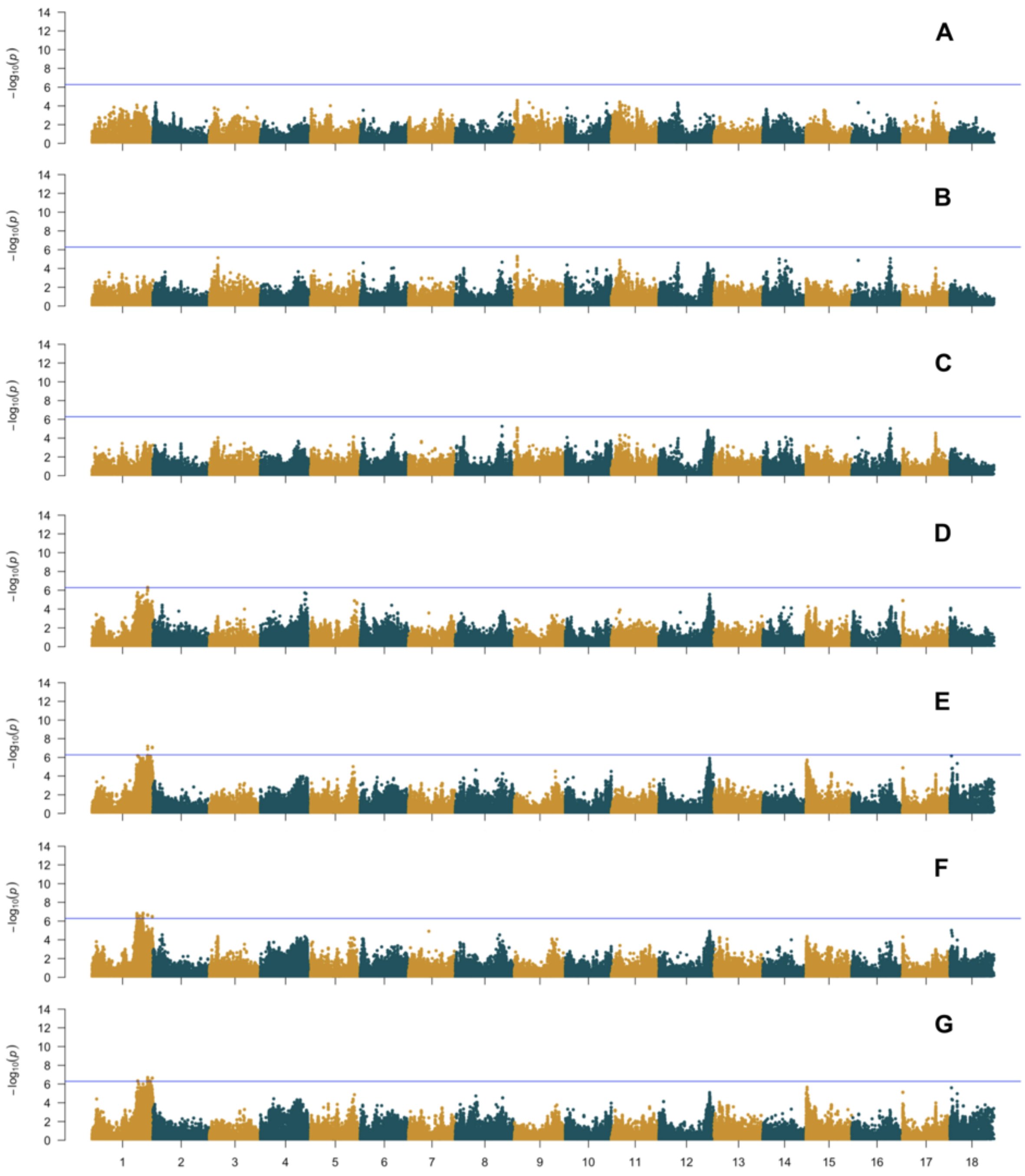

Supplementary figure 4. Size and shape GWAS results using the IITA Genetic Gain and Cycle 1 breeding populations using CMD score as a covariate. Manhattan plots of $-\log_{10}$(P-value) of A. Area; B. Perimeter; C. Feret; D.Solidity; E. Aspect ratio; F. Circularity; G. Roundness. Blue horizontal line indicates the Bonferroni statistical threshold.

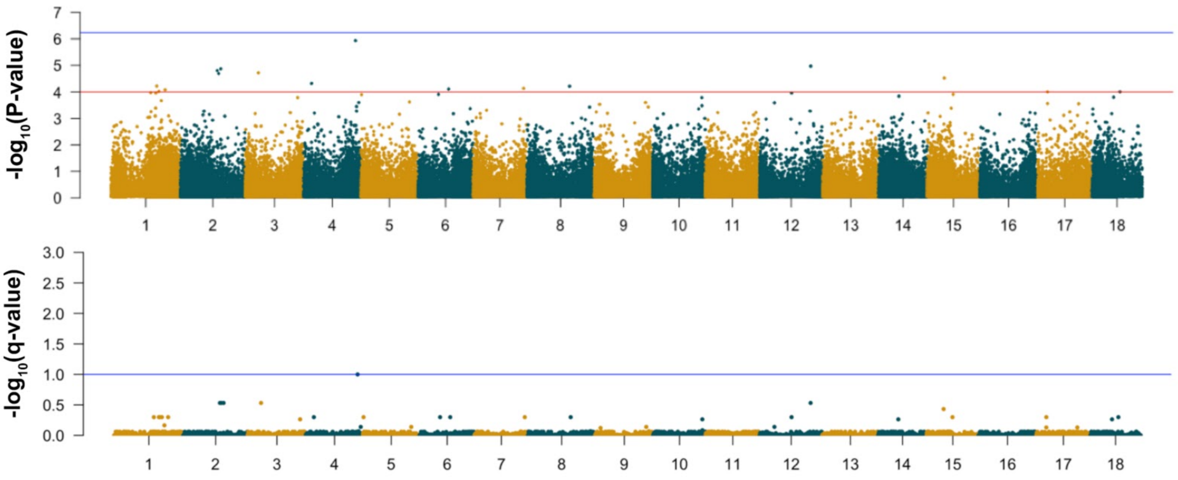

Supplementary Figure 5. Multivariate GWAS of Circularity, Round and Solidity (model 1). Manhattan plots of -log$_{10}$(P-value) (top panel) and -log$_{10}$(q-value) (bottom panel). In the top panel the blue horizontal line indicates the Bonferroni statistical threshold and the red line indicate a -log$_{10}$(p-value) = 4. In the bottom panel de the blue line indicates the significant threshold of the q-value.

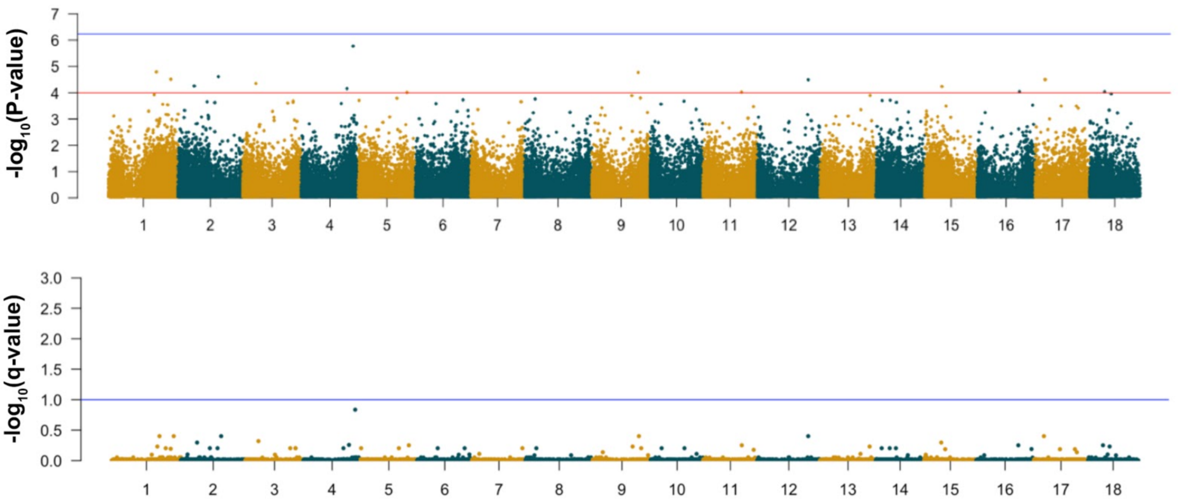

Supplementary Figure 6. Multivariate GWAS of Area, Feret and Circularity (model 2). Manhattan plots of -log$_{10}$(P-value) (top panel) and -log$_{10}$(q-value) (bottom panel). In the top panel the blue horizontal line indicates the Bonferroni statistical threshold and the red line indicate a -log$_{10}$(p-value) = 4.In the bottom panel de the blue line indicates the significant threshold of the q-value.

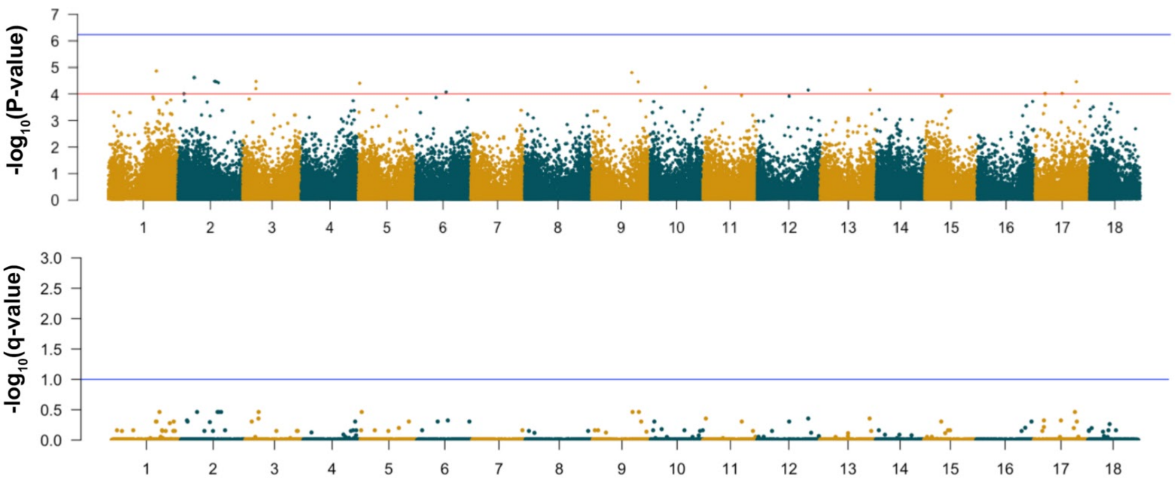

Supplementary Figure 7. Multivariate GWAS of Area, Perimeter, Round, Solidity and AR (model 3). Manhattan plots of -$\log_{10}$(P-value) (top panel) and -$\log_{10}$(q-value) (bottom panel). In the top panel the blue horizontal line indicates the Bonferroni statistical threshold and the red line indicate a -$\log_{10}$(p-value) = 4. In the bottom panel de the blue line indicates the significant threshold of the q-value.

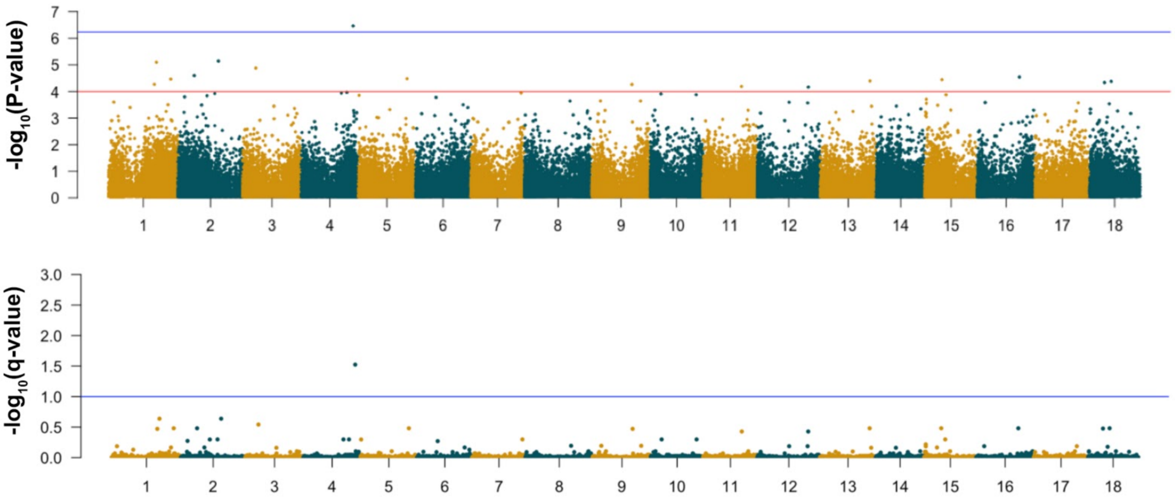

Supplementary Figure 8. Multivariate GWAS of Area, Perimeter, Feret, Circularity, Round, Solidity and AR (Model 4). Manhattan plots of -$\log_{10}$(P-value) (top panel) and -$\log_{10}$(q-value) (bottom panel). In the top panel the blue horizontal line indicates the Bonferroni statistical threshold and the red line indicate a -$\log_{10}$(p-value) = 4. In the bottom panel de the blue line indicates the significant threshold of the q-value.

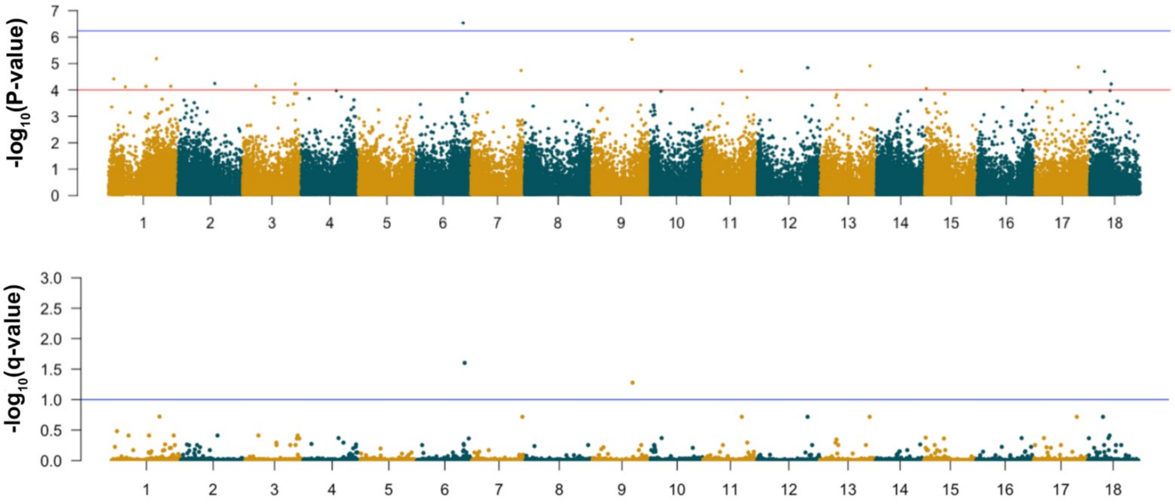

Supplementary Figure 9. Multivariate GWAS of Area, Perimeter and Feret (model 5). Manhattan plots of -log$_{10}$(P-value) (top panel) and -log$_{10}$(q-value) (bottom panel). In the top panel the blue horizontal line indicates the Bonferroni statistical threshold and the red line indicate a -log$_{10}$(p-value) = 4. In the bottom panel de the blue line indicates the significant threshold of the q-value.

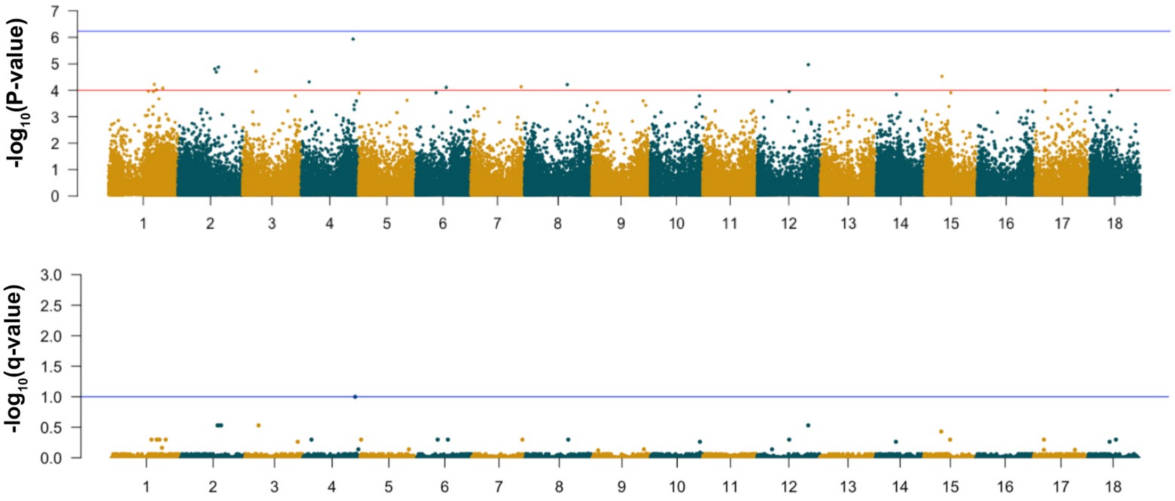

Supplementary Figure 10. Multivariate GWAS of Circularity, Round, Solidity and AR (model 6). Manhattan plots of -log$_{10}$(P-value) (top panel) and -log$_{10}$(q-value) (bottom panel). In the top panel the blue horizontal line indicates the Bonferroni statistical threshold and the

red line indicate a $-\log_{10}$(p-value) = 4. In the bottom panel de the blue line indicates the significant threshold of the q-value.
